# Replacing the projected retiring baby boomer nursing cohort 2001 – 2026

**DOI:** 10.1186/1472-6963-7-87

**Published:** 2007-06-16

**Authors:** Deborah J Schofield

**Affiliations:** 1 Northern Rivers University Department of Rural Health (Lismore), Faculty of Medicine, University of Sydney, Sydney, Australia

## Abstract

**Background:**

The nursing population in Australia is ageing. However, there is little information on the rate and timing of nursing retirement.

**Methods:**

Specifically designed health workforce extracts from the Australian Bureau of Statistics (ABS) censuses from 1986 to 2001 are used to estimate the rate of nursing retirement. The 2001 nursing data are then "aged" and retirement of the nursing workforce projected through to 2026. ABS population projections are used to examine the future age structure of the population and the growth and age distribution of the pool of labour from which future nurses will be drawn.

**Results:**

Attrition rates for nurses aged 45 and over are projected to be significantly higher between the base year of 2006 and 2026, than they were between 1986 and 2001 (p < 0.001).

Between 2006 and 2026 the growth in the labour force aged 20 to 64 is projected to slow from 7.5 per cent every five years to about 2 per cent, and over half of that growth will be in the 50 to 64 year age group. Over this period Australia is projected to lose almost 60 per cent of the current nursing workforce to retirement, an average of 14 per cent of the nursing workforce every five years and a total of about 90,000 nurses.

**Conclusion:**

The next 20 years will see a large number of nursing vacancies due to retirement, with ageing already impacting on the structure of the nursing workforce. Retirement income policies are likely to be a key driver in the retirement rate of nurses, with some recent changes in Australia having some potential to slow retirement of nurses before the age of 60 years. However, if current trends continue, Australia can expect to have substantially fewer nurses than it needs in 2026.

## Background

In Australia, two major government reports, the Intergenerational Report [1] and the Report on the Economic Implications of an Ageing Australia [2] have identified future budget pressures as a threat to the sustainability of the Commonwealth budget and the availability of health services to future generations. One of the main drivers was found to be growth in health expenditure. However, the shrinking supply of labour as the health workforce ages and retires may pose just as large a threat to the availability of health services.

There is no doubt that the health workforce is ageing [3,4] however, the timing of potential shortages depend upon when health professionals choose to retire. Nurses are the largest health professional group and, in the past in Australia, they have retired significantly earlier than medical practitioners [5]. As the oldest of the large baby boomer cohort turns 60 this year we can expect nursing retirement in significant numbers over the next ten to fifteen years.

However, there are few papers from Australia or other countries which project the rate or timing of nursing retirement [6,7] although a number have indicated that nursing shortages are anticipated as the nursing population ages [8-10]. At the same time as a large proportion of the nursing workforce will be entering retirement, projected growth in the size of the labour market is expected to slow [1]. Understanding the timing and extent of nursing retirement is particularly important as there is already a shortage of nurses in all Australian States and Territories – listed on the National Skill Shortage List released annually by the Department of Education, Science and Training [11].

This paper used a specially defined extract from the past four Australian Bureau of Statistics (ABS) full Censuses for the years 1986, 1991, 1996 and 2001 to measure and then project future nursing retirement through to 2026 and ABS population projections to estimate the size of the labour pool from which the future workforce will be drawn.

## Methods

Australian Bureau of Statistics (ABS) population estimates and projections were obtained to 2025 by single year of age and by sex [12,13]. The series used was the medium growth Series B, which assumes medium population growth resulting from medium migration, life expectancy and fertility. (Series A assumes high population growth as a result of higher migration, life expectancy and fertility and Series C assumes low population growth and has the same life expectancy and fertility assumptions as series B but lower migration[20].)

The population estimates were used to prepare population pyramids to examine ageing of the general population and the size and age distribution of the pool of labour from which future nurses will be drawn.

Attrition rates as nurses leave the workforce (due to factors including relocation, retirement, ill health and death) were estimated from the special extracts from ABS Census data (drawn from the full national data set) for registered nurses in 5-year age groups for the years 1986, 1991, 1996 and 2001. This Census data is not generally available as information on nurses is generally grouped into a single "health professional" occupation category. Attrition rates for nurses aged 45 to 75 and over were derived by following five-year cohorts through each of the 1986, 1991, 1996 and 2001 censuses and calculating the net loss from each cohort every five years. The formula is expressed as:

CAR = 1 - Nti/Nt1

where CAR= Cumulative attrition rate, N = number of people, ti=census date and t1=first census in series (1986).

The nursing population in 2001 was "aged" so that for every five years in the future, each five year age group moved up to the age group five years older to represent the nursing population in 5, 10, 15, 20 and 25 years time. The attrition estimates were applied every five years to estimate the net number of nurses aged 45 years who would leave the workforce as they aged. Attrition from 2001 to 2006 was estimated to establish a current base year for projections from 2006 to 2026.

It was assumed that past patterns of attrition within each five year age group would be maintained into the future and that between the ages of 75 and 80 all remaining nurses retired. This was a reasonable assumption as there were very small numbers in the 75+ age group. Sensitivity analysis was undertaken to determine the effect of a higher or lower rate of retirement than that projected.

Statistical analysis was undertaken using SAS (version 9.1). All tests were undertaken at the 5% level of significance. Tests of association between categorical variables were done using chi-squared tests. Although the data was from a full national census, tests were undertaken to determine whether differences over time within the nursing workforce were of a sufficient magnitude to be statistically significant.

## Results

### Population change

**Figure 1:**
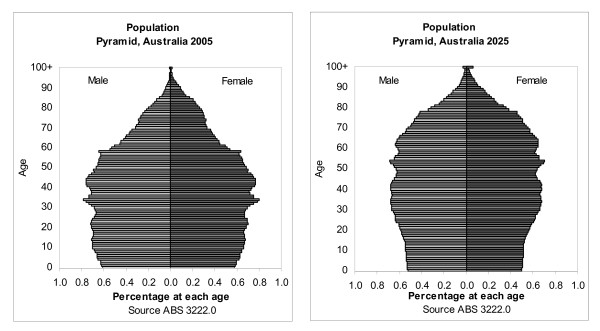
**Population Change, Australia, 2005 to 2025**. Source: ABS 2005a.

Over the next 20 years, the age structure of the population will change markedly as the large baby boomer cohort moves towards retirement and old age (figure 1). In 2005, the baby boomer bulge from about age 40 to 60 years was visible and the population pyramid tapers away quickly from the age of 60 years. However, by 2025 the population pyramid narrows at the base and expands at the top as the population ages, longevity increases and fertility declines. Population growth for persons less than 60 years of age is projected to be only 12% compared to 85% for persons 60 and over.

### Nursing ageing and retirement

There were a total of about 133,000 registered nurses in 1986 and 160,000 by 2001 (table 1). The nursing workforce was older in 2001 than it was in 1986. The proportion of nurses aged 40 years or more has more than doubled, and the proportion of nurses aged less than 40 years has fallen from about 70 to 40%.

**Table 1:** Nursing population by age, 1986 – 2001

Year	20–24	25–29	30–34	35–39	40–44	45–49	50–54	55–59	60–64	65–69	70–74	75+	Total
1986	21933	26474	22630	20214	15363	11625	7777	4995	1785	364	101	96	133357
1991	13494	20949	25916	24239	20705	14819	10221	5631	2469	401	96	45	138985
1996	12005	19109	21010	27550	26119	21279	13515	7635	2648	584	127	69	151650
2001	8123	16579	19073	22504	29620	26780	20382	11347	4708	1079	206	265	160666

Net attrition from the nursing population between one five year period and the next (derived by following five-year cohorts through each of the 1986, 1991, 1996 and 2001 censuses) appeared to begin as nurses moved from age group 45–49 to 50–54 with a net 12 per cent of nurses leaving the workforce (table 2). After 10 years, there was 38 per cent net attrition and after 15 years 60 per cent.

**Table 2:** Cumulative rates of retirement of nurses from 1986

Number of years later	Age at start year						
	
	45–49	50–54	55–59	60–64	65–69	70–74	75+
5	12%	28%	51%	78%	74%	55%	100%
10	34%	66%	88%	93%	81%	100%	100%
15	60%	86%	96%	100%	100%	100%	100%
20	78%	88%	100%	100%	100%	100%	100%
25	93%	96%	100%	100%	100%	100%	100%

Source: Health workforce supertable extract from ABS Census 1986, 1991, 1996 and 2001, authors' calculations

The projections of nursing retirement indicate that Australia can expect to need to replace an average of 14 per cent of the nursing workforce every five years from 2006 to 2026 due to older nurses leaving the workforce alone (table 3). This is between 20,000 and 25,000 nurses every five years and is a total of 90,200 between 2006 and 2026. Sensitivity analysis was undertaken to determine the effect of a higher or lower rate of retirement than that projected. If nurses were to retire at a rate of 1 percentage point faster for every 5 years over the projection period then a total of 89,500 nurses were projected to retire between 2006 and 2026 and 90,900 if the retirement rate were 1 percentage point slower every 5 years. The effect is less than 4% in total as when attrition reaches 100% the rate can be neither raised nor lowered.

**Table 3:** Nursing retirement 1986 to 2006 and forecast 2006 to 2026

Base year 2006	Number in each period	Cumulative retirements	% of workforce in base year
2006–11	22529	22529	14
2011–16	19567	42096	26
2016–21	22899	64995	40
2021–26	25223	90218	56
			
Base year 1986			
1986–91	7880	7880	7
1991–96	9104	16984	15
1996–01	7870	24854	22
2001–06	19421	44275	40

By contrast to the rapid attrition from 2006 onwards, between 1986 and 2001, only about a net 7 per cent of nurses over the age of 45 years left the workforce every 5 years (table 3). A comparison of the total number of nurses 45 years and over who left the workforce indicates that retirement rates will be significantly higher between 2006 and 2026 than they were between 1986 and 2001 and (p < 0.001). Between 2001 and 2006 however, there was estimated to be a net loss of 17 per cent due to older nurses leaving the workforce. About half of this larger figure is due to the oldest of the baby boomers aged about 55 to 60 years beginning to retire. This indicates that we have already entered a period of rapid retirement of older nurses from the workforce.

The large baby boomer cohort aged about 40 to 60 years made up 60 per cent of the nursing workforce in the census of 2001. A further 13 per cent are from older cohorts. The more rapid rate of attrition in the forecast from 2006 to 2026 than in 1986 to 2006 results largely from the movement of the baby boomer cohort, then aged about 40 to 60 in 2006, out of the workforce.

### Labour supply

**Figure 2:**
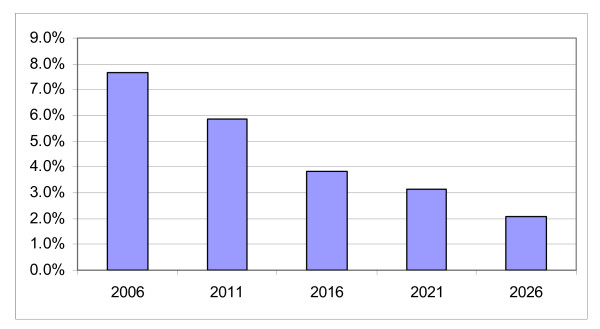
**Growth in working age population 20–64 years, Australia, 2006 to 2026**. Source: ABS population projections.

At the same time as the nursing workforce will age and a large proportion will be retiring, the growth in the Australian labour market will slow rapidly as the general population also ages (figure 2). Between 2006 and 2026 the growth in the labour force aged 20 to 64 will slow from about 7.5 per cent every 5 years to about 2 per cent. Prior to 2006 growth in the labour force was even higher, up to about 10 per cent for some five year periods in the 1990s and 1980s.

Further compounding the shrinking pool of labour from which to hire new nurses is the age groups in which the highest rates of labour growth will occur. 28 per cent of total growth from 2006 to 2026 will come from the 60–64 year age group and 56 per cent from the 50 to 64 age groups – the age groups from which nurses are more likely to be retiring than entering the labour force. This means that even as the growth in the labour force slows to less than a third of its current rate, less than half the growth in the labour force is likely to form part of the pool of labour from which nurses can be drawn to replace retiring nurses.

### Nursing labour supply and demand

From 2006 to 2026, the total population is expected to grow by 24% and the population aged 65 years or more by 79%. This growth in the aged population, even when offset by current policies which reduce hospital stay, has been projected to result in an increased demand for hospital bed days of about 40% from 2005 to 2025.

However, the population aged less than 30 years (when the majority of nursing students commence study) is only projected to grow by 8%. According to the Department of Employment, Training and Youth Affairs [19], approximately 8,000 students commenced a nursing degree each year and about 4,800 of those could be expected to complete within four years. By 2025, if the same proportion of the population under 30 years of age chose nursing as a career, there would be approximately, 5,100 completions per annum or about 25,800 completions in the five years to 2025. These students would fill the roughly 20,000 to 25,000 positions vacated by retiring nurses. However, this number of new nurses would not be adequate to also provide for the increased demand of a more aged population or the attrition from nursing that occurs prior to the age of 45 years.

## Discussion – the role of retirement income policy

Why is it that we have entered this rapid period of nursing retirement which is set to continue to 2026? Ageing of the population is part of the explanation, with about 75 per cent of the nursing population in the baby boomer cohort or older. Further explanations from Australia and other countries include a decline in nursing undergraduate commencements [14], retention difficulties and the higher average age of new graduates from nursing programs. [15]

These are the demographic and social underpinnings of this phase of rapid retirement from 2001 to 2026. But there is a policy environment in Australia that promotes nursing retirement before the age of 60 years. Most baby boomer and older nurses are employed within the hospital system and have access to employer superannuation. Those employed by public hospitals will have been required to join the State Authorities Superannuation Scheme (SASS) or the State Superannuation Scheme (SSS). These are savings schemes contributed to by members and the employer. They provide a "defined benefit" which is an accrued multiple of the employee's final average salary. The SASS was introduced in 1988 and was closed to new members in 1992 [16]. Those who retire can take their benefits at their eligible retirement age of either 55 years (for members who transferred from older schemes) or 58 years. The SSS was introduced prior to the SASS, but was closed in 1988 when the SASS was introduced. Under the SSS, members elected a retirement age of 55 or 60 years and contributed their savings accordingly with higher contributions for those who chose the younger retirement age. However, it is possible for a nurse who elected retirement at 60 to retire earlier once they have reached 55 years, but their pension will be at a lower rate.

Nurses in these two schemes would be in the baby boomer or older cohorts now aged about 40 years or more and this helps explain the high rate of retirement between 55 and 60 years. It also suggests that the currently gradual increasing of age of eligibility for age pension for women in Australia from 60 years of age to 65 years of age and incentives to delay retirement [17] may not result in high workforce participation of older nurses.

Increasing the average retirement age of nurses would go some way to alleviating future workforce shortages. Biviano et al [6] indicates that delaying nursing retirement by 4 years has a modest effect (9 per cent in 2020) on increasing nursing supply in projections of nursing supply for the US.

Until recently, retirees have not been able to draw a pension and continue to work part time which has been a disincentive to retire partially rather than completely. However, newly introduced changes to superannuation in Australia will provide more incentive for nurses and other Australians to keep working. The Australian Government have changed the superannuation rules to allow workers to continue to work while drawing on a superannuation pension from 1 July 2005 [18]. This may encourage nurses, and particularly women with children with a lower accumulated balance because of broken employment patterns, to continue to work. However it remains to be seen whether this economic incentive will be sufficient to persuade nurses to work longer as there are other non-economic reasons why nurses may have been retiring early, including musculoskeletal injuries and the heavy physical nature of nursing [21], job characteristics such as inflexibility of shift work, working conditions and relatively low pay for the skills required [22] and, for women, aligning their retirement age with on average older husbands [5,23].

## Conclusion

Australia faces a period of rapid retirement from the nursing workforce which has recently begun and which will mean the loss of about 90,000 nurses over the next 20 years. Ageing of the population and population growth will result in increased demand for nursing care that is unlikely to be met by the current proportion of young adults completing nursing training. Careful planning to balance recruitment of new nurses, and policy incentives to improve retention of nurses is required to ensure that there is an adequate nursing workforce to meet the needs of an ageing population.

## Competing interests

The author(s) declare that they have no competing interests.

## Authors' contributions

DS undertook the analysis and prepared the paper.

**Table 4:** Growth in the labour market age 20–64, Australia, 2006 to 2026

Growth	20–24	25–29	30–34	35–39	40–44	45–49	50–54	55–59	60–64
N	48470	170557	185616	167606	178361	117278	262376	299401	543620
% for age group	3%	12%	12%	11%	12%	8%	19%	23%	55%
% of total growth	2%	9%	9%	8%	9%	6%	13%	15%	28%

## Pre-publication history

The pre-publication history for this paper can be accessed here:


